# Expansion of Microsatellites on Evolutionary Young Y Chromosome

**DOI:** 10.1371/journal.pone.0045519

**Published:** 2013-01-16

**Authors:** Eduard Kejnovský, Monika Michalovova, Pavlina Steflova, Iva Kejnovska, Susana Manzano, Roman Hobza, Zdenek Kubat, Jan Kovarik, Manuel Jamilena, Boris Vyskot

**Affiliations:** 1 Department of Plant Developmental Genetics, Institute of Biophysics, Academy of Sciences of the Czech Republic, Brno, Czech Republic; 2 Laboratory of Genome Dynamics, CEITEC - Central European Institute of Technology, Brno, Czech Republic; 3 Department of CD Spectroscopy of Nucleic Acids, Institute of Biophysics, Academy of Sciences of the Czech Republic, Brno, Czech Republic; 4 Departamento de Biologia Aplicada, Escuela Polytecnica Superior Universidad de Almeria, Almeria, Spain; 5 Laboratory of Molecular Cytogenetics and Cytometry, Centre of the Region Haná for Biotechnological and Agricultural Research Institute of Experimental Botany, Olomouc, Czech Republic; University of Lausanne, Switzerland

## Abstract

Sex chromosomes are an ideal system to study processes connected with suppressed recombination. We found evidence of microsatellite expansion, on the relatively young Y chromosome of the dioecious plant sorrel (*Rumex acetosa*, XY1Y2 system), but no such expansion on the more ancient Y chromosomes of liverwort (*Marchantia polymorpha*) and human. The most expanding motifs were AC and AAC, which also showed periodicity of array length, indicating the importance of beginnings and ends of arrays. Our data indicate that abundance of microsatellites in genomes depends on the inherent expansion potential of specific motifs, which could be related to their stability and ability to adopt unusual DNA conformations. We also found that the abundance of microsatellites is higher in the neighborhood of transposable elements (TEs) suggesting that microsatellites are probably targets for TE insertions. This evidence suggests that microsatellite expansion is an early event shaping the Y chromosome where this process is not opposed by recombination, while accumulation of TEs and chromosome shrinkage predominate later.

## Introduction

Microsatellites are short sequence motifs (1–6 bp) that are very common in eukaryotic genomes and are one of the most dynamic components of genome [1], [2]. The expansion of simple sequence repeats was common during formation of the first genomes and repetitive sequences make up a significant proportion of present-day genomes [3]. When cryptic simplicity, i.e. regions where simple repeats are over represented [4], is taken into account, two thirds of human genomic DNA was formed by microsatellites [5]. An expansion is a general property of most microsatellites. However, the mechanism of microsatellite expansion is far from being fully understood. One of the most well-established models of microsatellite expansion involves replication slippage [1].Quantitative experiments showed that the expansion potential is proportional to array length and inversely proportional to monomer length [6]. The expansion of microsatellite arrays is counteracted by point mutations that break perfect repeats. The substitution rate is highest at the ends of array [7]. Erosion of arrays then results in cryptic simplicity of flanking sequences.

Many microsatellite motifs can adopt unusual DNA conformations such as hairpins, quadruplexes, parallel duplexes and even left-handed Z-DNA [8]–[10]. Several decades ago it was shown that poly(dC-dA).poly(dT-dG) motifs can adopt a Z-DNA conformation [11]. Most (if not all) non-B-DNA conformations appear to play a role in genome rearrangements [12]. AC/GT repeats are associated with regions of high recombination, although it is not clear whether microsatellites are the cause or consequence of high recombination rates. Majewski and Ott [13] proposed that microsatellites may be involved in the early stages of recombination, especially when forming longer repeat arrays. There is experimental evidence that the Z-DNA conformation of the CA/TG motif promotes homologous recombination in human cell cultures [14], [15].

Sex chromosomes are good model systems for the study of specific evolutionary processes that occur when recombination is suppressed. The non-recombining parts of the Y chromosome are unique genomic regions where processes of gene degeneration and repeat accumulation, including microsatellites, are active [16]. In *Drosophila miranda*, reduced level of microsatellite variability was found on the neo-Y chromosome [17]. In the dioecious plant *Silene latifolia,* non-recombining regions of the evolutionarily young Y chromosome [16] have accumulated repetitive DNA [18], [19] including most of mono-, di-, and trinucleotide microsatellites [20].

Microsatellites are sometimes associated (colocalized) with other genomic repeats, especially transposable elements. In humans, microsatellites are associated with repetitive DNA, especially non-LTR retrotransposons [21], [22]. In plants including *Arabidopsis*, rice, soybean, maize, and wheat, however, microsatellites are preferentially located in non-repetitive DNA regions, which indicates that they reside in regions pre-dating genome expansion [23], [24]. The differences in microsatellite localization between mammals and plants can be attributed to the fact that mammalian genomes have large numbers of non-LTR retrotransposons, while LTR retrotransposons dominate in plant genomes. It was hypothesized that close association of microsatellites with non-LTR retrotransposons like SINE elements in mammalian genomes is result from insertion of polyadenylated SINE retrotranscripts, followed by mutations expanding their poly(A) sequences [25]–[27]. It is also possible that transposable elements, especially non-LTR retrotransposons, whose movement involves target-primed reverse transcription (TPRT), preferentially insert into microsatellite arrays [25].

In this study we show that microsatellites expanded on the relatively young and large Y chromosomes of *Rumex acetosa* (Y_1_Y_2_X system, 12–13 mya) [28] but not on the older and small Y chromosome of human (XY system, 150 mya) [29] or *Marchantia polymorpha* (XY system) [30]. The expansion mechanism is likely to be reflected in the length periodicity of microsatellite arrays, and we propose that DNA conformation is an important factor. Our data also indicate that microsatellite arrays are targets for transposable element insertions.

## Results

### FISH Analysis of Microsatellite Accumulation on Both Y Chromosomes in *R. Acetosa*

**Figure 1:**
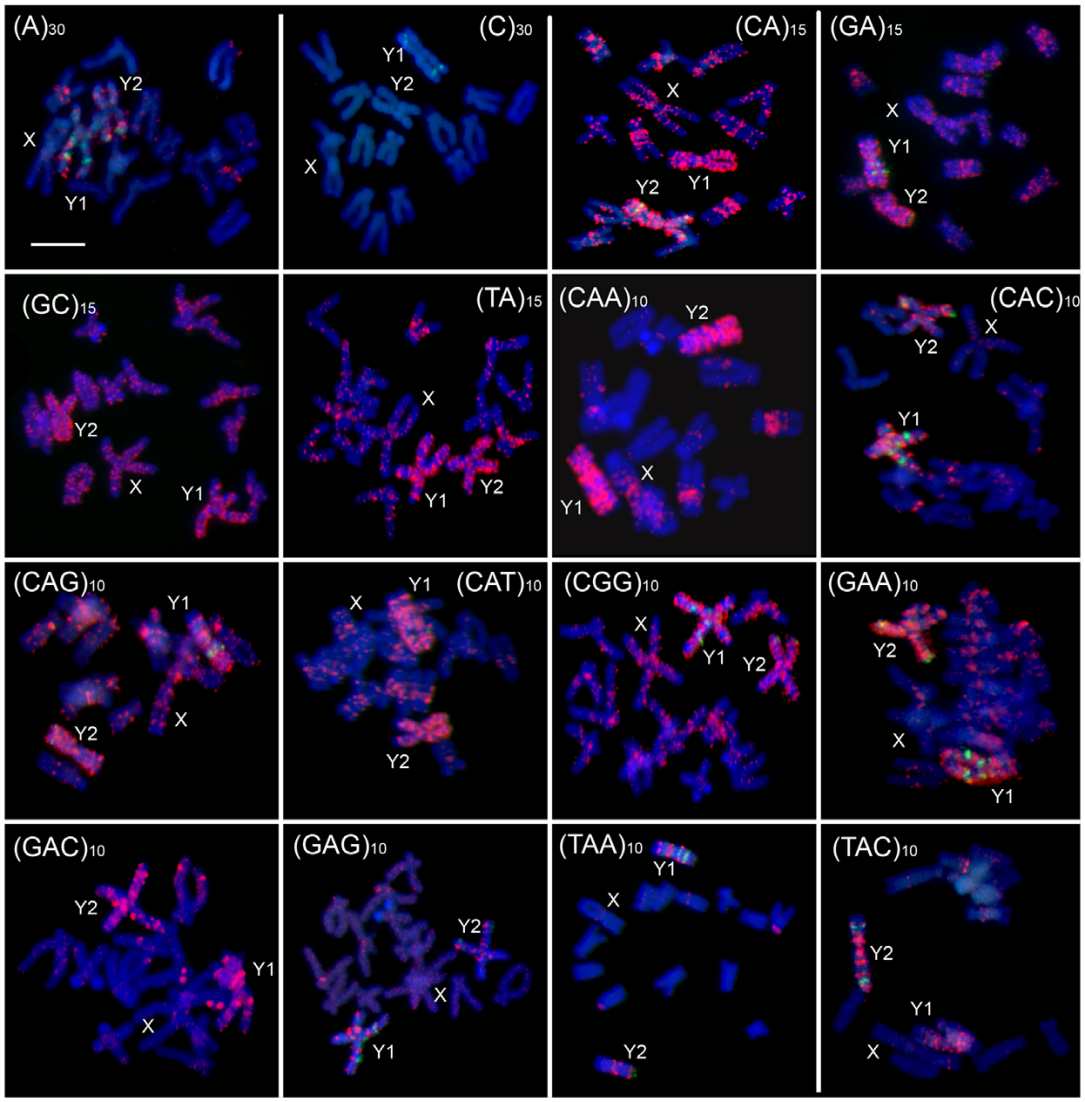
Fluorescent *in situ* hybridization of mitotic metaphase chromosomes of male *Rumex acetosa* hybridized with various labeled microsatellite-containing oligonucleotides as indicated. Chromosomes were counterstained with DAPI (blue); microsatellite probes were directly labeled with Cy3 during synthesis (red signals). The cytogenetic marker RAYSI (green) was used to identify Y1 and Y2 chromosomes. The X and Y chromosomes are indicated. The bar indicates 10 µm.

We used fluorescent *in situ* hybridization (FISH) to study the chromosomal distribution of all possible mono-, di- and tri-nucleotide microsatellites (taking into account permutations of these microsatellites and permutations of their complementary strands) in *Rumex acetosa*. We used following labeled oligonucleotides - d(A)_30_, d(C)_30_, d(CA)_15_, d(GA)_15_, d(GC)_15_, d(TA)_15_, d(CAA)_10_, d(CAG)_10_, d(CGG)_10_, d(GAA)_10_, d(CAC)_10_, d(CAT)_10_, d(GAC)_10_, d(GAG)_10_, d(TAA)_10,_ d(TAC)_10_. Most of these microsatellites showed a strong signal of accumulation on the Y1 and Y2 chromosomes with weaker signal on other chromosomes (Figure 1). Only (C)_30_ did not show a detectable signal on any chromosomes. The strongest signal of accumulation on the Y chromosomes was exhibited by the (CAA)_10_, (GAA)_10_, (CA)_15_, (TAC)_10_ and (GA)_15_ microsatellites. These probes painted the entirety of both the Y1 and Y2 chromosomes, while other probes exhibited discrete signals along both Y chromosomes. The extent of microsatellite accumulation was similar on both the Y chromosomes suggesting they are of the same age and supporting the likelihood of a common origin by the splitting of an ancestral Y chromosome rather than translocation of autosome onto the X chromosome [31].

### Length of Microsatellite Arrays Revealed from Sequencing Data

**Figure 2:**
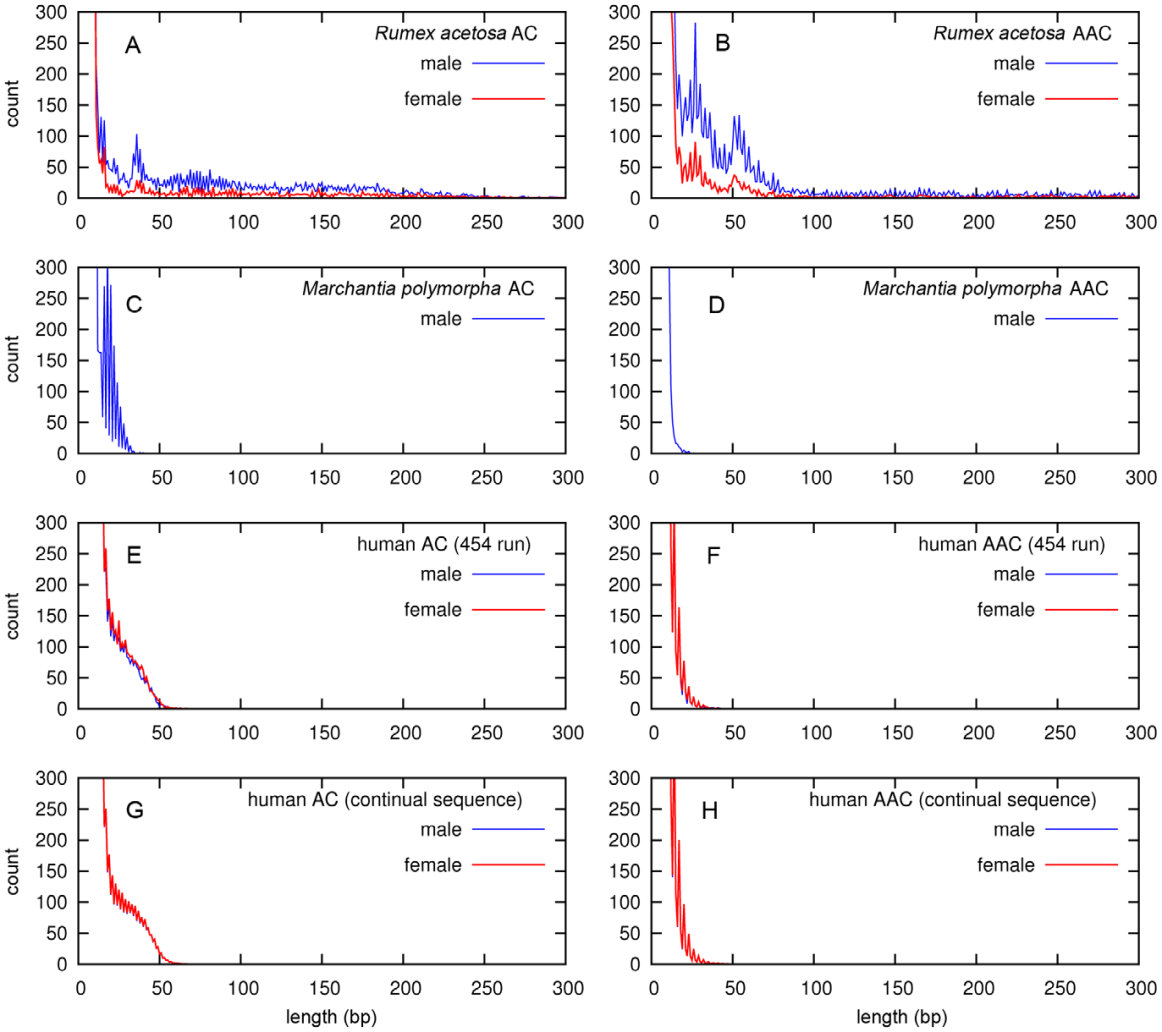
The length distribution of microsatellite arrays AC and AAC. The length of AC and AAC microsatellite arrays in *Rumex acetosa* (A and B, respectively), *Marchantia polymorpha* (C and D) and human (E–H) plotted against arrays abundance determined from 454 sequencing data (A–F) or continual sequence (G, H) calculated per 100 Mb. Males are in blue, females are in red.

**Figure 3:**
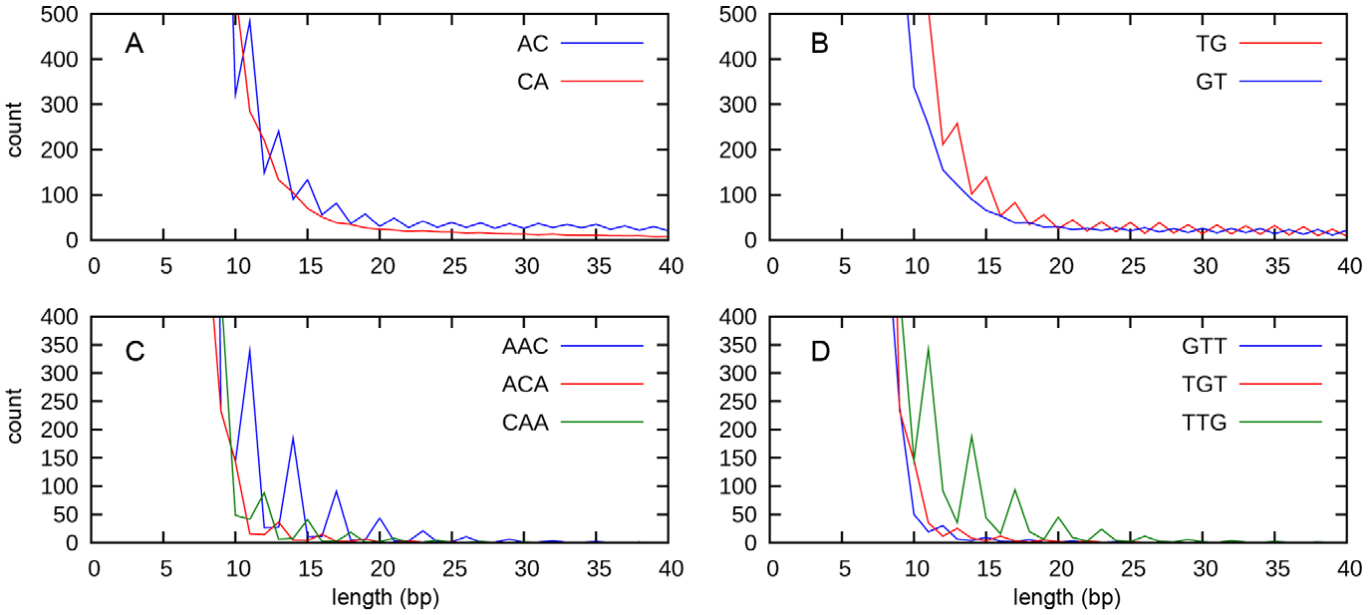
The length distribution of selected microsatellite arrays in human chromosomes. The analysis of all human chromosomes showing abundance of AC/CA (A), TG/GT (B), AAC/ACA/CAA (C) and GTT/TGT/TTG (D) microsatellite arrays plotted against their length.

For our analysis of microsatellites, we used 454 sequencing data obtained for *Rumex acetosa* male and female plants. Additionally, we used human male and female 454 sequencing data from The 1000 Genomes Project [46] database as well as *Marchantia polymorpha* male 454 data (female was not available). In parallel, we analyzed continual human sequence from GeneBank – male (autosomes+X+Y) and female (autosomes+X). We estimated the numbers of microsatellite arrays of given length for all possible mono-, di- and trinucleotide microsatellites. Most of our analyses considered all possible permutations in both complementary strands as a single microsatellite type, e.g., type AAC included AAC, ACA, CAA, TTG, TGT and GTT microsatellites (as in Figures 2, S1, S2, S3, S4). In some analyses, we studied complementary strands separately (as in Figure 3). Two patterns can be observed in the plots of individual microsatellite array length (Figure 2A, B, S1) – the expansion of microsatellite arrays and the differences between male and female of *R. acetosa*. The longest arrays were formed by AC, and AAC microsatellites, which reached the lengths of 250 bp (Figures 2 A, B, S1). Most microsatellite motifs showed the similar length and repeat numbers in both sexes (Figure S1), but AAC and AC microsatellites in *R. acetosa* have higher repeat numbers in male than in female (Figures 2A, B). In *Marchantia polymorpha*, where Y chromosome is smaller than X chromosome and sex chromosomes are probably older [30], no expansion of microsatellites was observed (Figure 2C, D, S2). Similarly, in human – both in 454 data and in continual sequence - the AC and AAC microsatellites were expanded compared to other motifs, but the lengths were only 21–50 bp (Figures 2, S3, ), and we found no length differences between human male and female (Figure 2E–H).

Microsatellite arrays (loci) present in the genome could be longer than 454 sequencing reads and some reads can consist entirely of microsatellites, and will under-represent the complete length. We therefore checked the abundance of arrays of given lengths for individual microsatellites in *R. acetosa*, where microsatellite arrays were longest, by comparing all microsatellite arrays and only internally located microsatellite arrays (Figure S5). Reads ending with microsatellites sequence, or reads entirely made up of microsatellite sequence, are a significant proportion of AC and AAC microsatellites in *R. acetosa*. This suggests that AC and AAC form long genomic arrays, consistent with the strong FISH signal of AC and AAC microsatellites, and their accumulation on the *R. acetosa* Y chromosomes (Figure 1).

### Length Periodicity of Microsatellite Arrays

The AC and AAC microsatellites display a remarkable periodic pattern, especially in *R. acetosa* (Figure 2A, B) and *M. polymorpha* (Figure 2C, D). The majority ofl permutations of both strands – AC, CA, TG and GT as well as AAC, ACA, CAA, GTT, TGT and TTG – showed this pattern. The periodic pattern of AC and AAC arrays could be explained either by differences in abundance of arrays of specific lengths, or as artifacts caused by nonrandom fragmentation of microsatellite arrays at specific di- or tri-nucleotides, or by artificial stepwise expansion during 454 sequencing when emulsion PCR amplification is used. To exclude the possibility of sequencing artifacts, we analyzed continual DNA sequence from all human chromosomes that were generated by classical sequencing methods and found the AC and AAC periodicity with AC and AAC with peaks shifted by one base for each permutation in one strand suggesting the importance of terminal di- or trinucleotides (Figure 3). For example, the 13-mer starting with ACA, the 14-mer starting with AAC and the 15-mer starting with CAA, all end with the CAA motif (Figure 3C).

To find the most abundant starting and ending sequences, we calculated all possible beginnings and ends of microsatellite arrays in periodic regions (e.g. 11–31 bp) for AC and AAC motifs. We found a strong over-representation of certain start and end motifs (Table 1). The AAC/ACA/CAA/TTG/TGT/GTT arrays preferentially started with AAC and ended with the CAA motif or started with TTG and ended with GTT. Similarly, for dinucleotides, the most frequent starts were AC and TG, while the most common ends were CA and GT. It is notable that the most abundant arrays were complementary i.e. AAC(AAC)nAA and TTG(TTG)nTT as well as AC(AC)nA and TG(TG)nT.

**Table 1:** The abundance of AC and AAC microsatellite arrays with specific beginning and ending sequences in male *R. acetosa* 454 data, male *Marchantia polymorpha* 454 data, male human 454 data and human continuous sequence.

			RUMEX male	MARCHANTIA male	HUMAN male (454)	HUMAN male
			INTERNALLY	INTERNALLY	INTERNALLY	ALL
	beginning	end	L: 11–31	L: 11–31	L: 11–31	L: 11–31
**(AAC)n**	AAC	AAC	155	82	150	1,452
**(AAC)n A**	AAC	ACA	135	41	156	1,512
**(AAC)n AA**	AAC	CAA	**637**	**323**	**1,979**	**21,662**
**(ACA)n AC**	ACA	AAC	115	177	41	416
**(ACA)n**	ACA	ACA	160	71	41	443
**(ACA)n A**	ACA	CAA	101	57	188	1,969
**(CAA)n C**	CAA	AAC	62	49	25	247
**(CAA)n CA**	CAA	ACA	252	234	141	1,418
**(CAA)n**	CAA	CAA	142	139	475	5,112
**(GTT)n**	GTT	GTT	89	96	143	1,483
**(GTT)n GT**	GTT	TGT	129	159	48	430
**(GTT)n G**	GTT	TTG	56	73	34	268
**(TGT)n T**	TGT	GTT	103	51	142	1,498
**(TGT)n**	TGT	TGT	288	70	46	423
**(TGT)n TG**	TGT	TTG	192	267	151	1,351
**(TTG)n TT**	TTG	GTT	**580**	**356**	**1,894**	**22,007**
**(TTG)n T**	TTG	TGT	292	40	170	1,880
**(TTG)n**	TTG	TTG	102	141	460	5,371
			**L: > = 14**	**L: > = 14**	**L: > = 14**	**L: > = 14**
**(AC)n**	AC	AC	126	3,313	1,223	15,477
**(AC)n A**	AC	CA	29	215	**1,912**	**24,551**
**(CA)n C**	CA	AC	21	135	483	6,808
**(CA)n**	CA	CA	14	106	1,000	11,622
**(GT)n**	GT	GT	193	2,872	1,154	15,543
**(GT)n G**	GT	TG	20	185	555	6,974
**(TG)n T**	TG	GT	**409**	172	**1,680**	**24,597**
**(TG)n**	TG	TG	65	67	916	11,631

The range of lengths of the arrays is always indicated.

The complementarity of AAC(AAC)nAA and TTG(TTG)nTT as well as AC(AC)nA and TG(TG)nT arrays suggests the possibility that microsatellites could play some role in long-distance DNA-DNA interactions during recombination or gene conversion. Therefore, we studied the distribution of these four microsatellite arrays along human chromosome 1 and compared it with the local recombination rate (see Material and Methods). We found that the abundance of microsatellite arrays and recombination rates are uniformly distributed along chromosome 1, with the exception of (peri)centromeric regions where both are significantly reduced (S6).

### Microsatellite Arrays are Expanded (not Multiplicated) on Young Y Chromosome

The accumulation of microsatellites in non-recombining regions of the Y chromosome could be caused either by microsatellite multiplication (duplications) or by increased repeat numbers of arrays. Therefore, to estimate the number of microsatellite loci, we counted the number of arrays formed by three and more monomers (Figure S7); short arrays were not visualized in Figure 2 and 3 because of their high count. We then calculated the number of microsatellite loci per unit length (100 Mbp). In *R. acetosa*, *M. polymorpha* and humans mononucleotide loci had the highest abundance followed by dinucleotide arrays and finally trinucleotide arrays (Figure S7). Among dinucleotide arrays, CG microsatellites were least abundant, while among trinucleotide ones, ACG and CCG showed the lowest abundance. In *R. acetosa*, the numbers of microsatellite loci were similar in males and females, suggesting that the higher number of long arrays (e.g. AC or AAC) in *R. acetosa* males than females (Figure S7A) probably results from microsatellite array expansion, rather than formation of new loci on the Y chromosome. The numbers were similar also in *M. polymorpha* male (Figure S7B) and human male and female (454 sequencing data, Figure S7C) as well as in human X and Y chromosomes (continual sequence, Figure S7D). Additionally, the similar relative abundances of most microsatellites among the three studied species probably reflect motif-specific expansion (Figure S7).

### Microsatellites Near Transposable Elements

**Figure 4:**
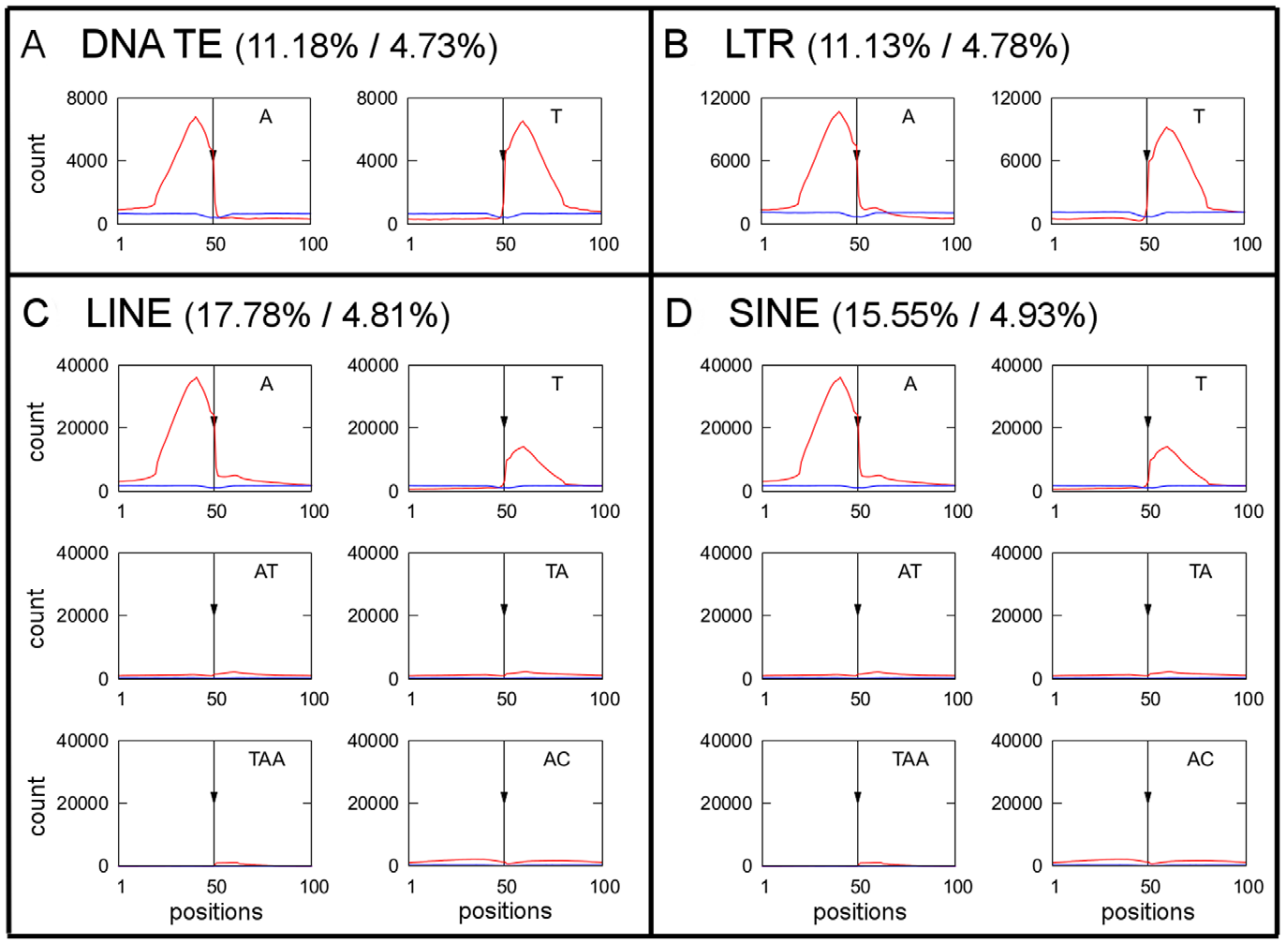
The occurrence of selected microsatellites near transposable elements. The occurrence of selected microsatellites in the 50 bp upstream (positions 1–50) and 50 bp downstream (positions 51–100) of transposable elements insertion sites (indicated by arrow) in DNA transposons (A), LTR retrotransposons (B), LINE (C) and SINE (D) in human. The observed distributions of microsatellites in the vicinity ofTEs are in red, distributions of microsatellites in a vicinity of random loci from our simulations are in blue (see Materials and Methods). Proportion of TEs (%)/proportion of random loci (%) that have at least one microsatellite array in their vicinity are indicated for each TE group.

To test the idea that microsatellites could be potential target sites for transposable element insertions, we analyzed the localization of transposable elements in the entire human genome. We measured the abundance of microsatellites of at least 10 bp (without allowing any sequence differences) located 50 bp upstream or downstream of transposable element insertion sites. In this analysis, we included transposable elements inserted in both sense and antisense strands. In Figure 4, we presented microsatellite distribution related to TEs (5′ end is always at left side and 3′ end at right side). Certain microsatellite types of each of the four TE types studied (LTR retrotransposons, LINEs, SINEs and DNA transposons) were enriched on both sides of TE insertion sites (Figure 4). The neighborhoods of DNA transposons and LTR retrotransposons were enriched with A microsatellites upstream of the insertion site, relative to the TE orientation, and T microsatellites downstream (Figure 4A, B). For LINEs and SINEs, situation was similar but additionaly AT, TA, TAA and AC were present downstream of element insertion sites to some extent (Figure 4C, D). At least one microsatellite array was present close to 11% of DNA transposons, 11% of LTR retrotransposons, 18% of LINEs and 16% of SINEs. In order to assess the significance of microsatellite-TE collocalization, in parallel we calculated the association of microsatellites with randomly chosen chromosomal loci. Our data show that enrichment of microsatellites in the vicinity of specific groups of transposable elements is 2,3 −3,7 times higher than would be expected for randomly chosen chromosomal loci (Figure 4).

### Induction of A-DNA and Left-handed Z-DNA in Most Expanding Microsatellites - AC and AAC

Motivated by the idea that the highest abundance of some motifs in genomes somehow reflects their DNA conformation, we used a set of AC and AAC-containing oligonucleotides (and their complementary strands) with different beginnings and ends and measured their ability to adopt an A-DNA form by circular dichroism (CD) spectroscopy. The transition is exemplified by (CA)_20_C+(GT)_20_G (Figure S8A) that most readily adopted A-DNA. Other oligonucleotides used in this study adopted A-DNA at slightly higher concentrations of TFE (Figure S8B). Additionally, we observed the transition of (CA)_20_C+(GT)_20_G as well as (AC)_20_A+(TG)_20_T to the left-handed Z-DNA conformation at increasing concentrations of CsF as indicated by the appearance of a negative band in the long wavelength part of the CD spectra (Figure S8C). In contrast, AAC microsatellites with different beginnings and ends did not produce negative band in long wavelength part of spectra (Figure S8D) what indicates that they did not adopt Z-DNA conformation even at higher concentrations of CsF (6.2M). The decrease of ellipticity at 277 nm at increasing CsF concentration is evident in AC-containing repeats but not in AAC-containing repeats (Figure S8E). The transition of all AC-containing dinucleotides and AAC-containing oligonucleotides to the A-DNA form and AC-containing oligonucleotides to the Z-DNA form, irrespective of their starting and ending motifs, suggests that the beginnings and ends were not critical for an ability to adopt either the A-DNA or Z-DNA conformations, at least in a case of the oligonucleotides used in this study.

## Discussion

**Figure 5:**
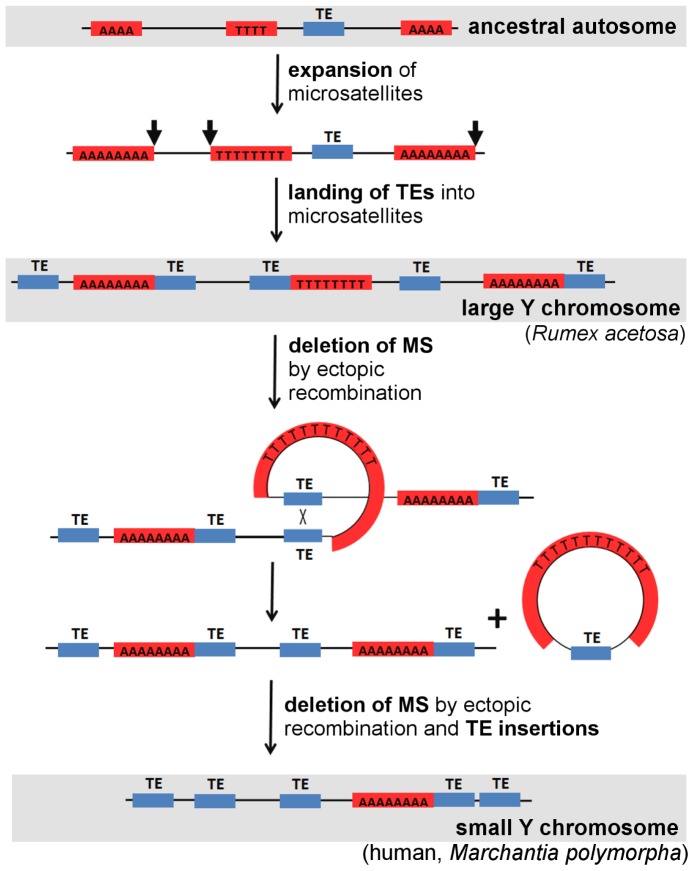
A model depicting the dynamism of microsatellites and transposable elements (TEs) on the evolving Y chromosome. The first event is an expansion of microsatellite arrays (red boxes) on a young Y chromosome. Then TEs (blue boxes) are inserted (black arrows) into microsatellite loci. Later ectopic recombination between homologous TEs results in deletions of intervening regions containing microsatellites. Finally, an old Y chromosome contains only short microsatellite loci (interrupted by TEs) and a large number of TEs.

We used two independent methods: fluorescent *in situ* hybridization (FISH) and next-generation 454 sequencing to show that some microsatellite motifs, like AC and AAC, have expanded on the young and large non-recombining Y chromosomes of *R. acetosa* (12–13 mya) [28]. This expansion is not visible on the old and small Y chromosomes of human (150 mya) [29] and *Marchantia polymorpha* [30]. We suggest that expansion ability is an intrinsic property of some microsatellites, and the non-recombining regions of the young Y chromosome provide only the opportunity for expansion [32], while the revitalizing process of recombination opposes this expansion on other chromosomes. Our finding that the number of total microsatellite loci was preserved during evolution of the Y chromosome in *R. acetosa*, but the arrays are extended in length supports this view. We therefore propose that early Y chromosome evolution is accompanied by microsatellite expansion (Figure 5). Our model can also explain sequence patterns on the older Y chromosome, which is characterized by short microsatellite arrays and higher number of transposable elements by proposing that microsatellites served as targets for TE insertions. Support for this aspect of the model is provided by our finding of colocalization of microsatellites with transposable elements, and by a similar phenomenon found by others in a partial human genome sequence [21].

Nadir et al [21] proposed that microsatellites can result from transposable element integration, but microsatellite arrays may also favor transposable elements insertion [21], [25]. Our data cannot definitively show whether microsatellites are the cause or the consequence of transposable element insertions. However, we prefer the latter view, for the following reasons. It is known that many transposable elements can target their insertion into preferred DNA sites [33]. Some transposases of DNA transposons and integrases of retrotransposons prefer bent DNA targets [34], [35], and DNA-bending proteins can help transposition [36]. It is therefore likely that transposable elements are able to recognize DNA conformation in addition to sequence. Microsatellites that are conformationally very flexible could be good targets for transposable element insertion. For example, high-resolution mapping of Sleeping Beauty DNA transposon integration in mammals showed a strong bias towards microsatellites [34]. The AA dinucleotide step exhibits the highest wedge angle, and A-tracts cause the strongest DNA bending [37], [38]. Our finding of enrichment of adenines upstream, and thymines downstream, of TE insertion sites in humans could support the preferential insertions into bent DNA. Similar mode was shown in Ty5 and Hermes transposons in *S. cerevisiae* that preferentially target nucleosome-free chromatin [39], [40]. Microsatellites might participate in TE targeting by opening the chromatin, perhaps by switching to an unusual DNA conformation (Z-DNA, A-DNA), into which transposons can then insert.

Our data showing remarkable periodicity in microsatellite arrays length and the preferential occurrence of specific beginnings and ends of microsatellite arrays could reflect the DNA extension mechanism, which we speculate could also be connected with DNA conformation [41]. Indeed, AC and AAC, the most expansion-prone motifs adopted an A-DNA form and the AC motif also adopted a left-handed Z-DNA conformation, in accord with previous studies [11]. We observed remarkable mutual complementarity of the most abundant arrays - AAC(AAC)nAA and TTG(TTG)nTT, and AC(AC)nA and TG(TG)nT. These sequences provide direct and inverted repeats with exact complementary beginnings and ends homogenously spread along the chromosomes. Such sequences could play a role in genome rearrangement, e.g. during recombination. Although like microsatellites, some transposable elements accumulate preferentially in regions with high recombination rates and possibly utilize recombination machinery for integration [42], many other repeats are gathered in regions with no or low recombination [32], [43]. Therefore, the chromosomal distribution of microsatellites and transposable elements is probably a result of various processes contributing to the repeat dynamism shaping both regions of high and low recombination.

Our model needs to be tested by analysis of microsatellites in other plant species with both large Y chromosome (e.g. *Silene latifolia*, *Coccinia grandis*, *Cannabis sativa*) and small Y chromosome (e.g. *Humulus lupulus*, *Cycas revoluta*) of different age [44] as well as animal models with reduced-size Y chromosome. The complex picture can be obtained by looking also at plants with homomorphic sex chromosomes (e.g. *Carica papaya*, *Bryonia dioica*) illustrating the stage before microsatellites started to expand. A remaining question is what extent and pattern of microsatellite accumulation can trigger a regressive phase of sex chromosome evolution. During a regressive phase the Y chromosome starts to shorten due to processes we suggested in our model (Figure 5). Since to our knowledge there are not many well characterized examples of such a phase of sex chromosome evolution we speculate that this process of shrinkage by large deletions is rapid. The loss of genetic material should have no effect on fitness because it is predated by degenerative processes caused by genetic and epigenetic activity of transposable elements that lower gene expression.

In conclusion, we contributed to a view that presents microsatellites as dynamic components of genome expansion, the signals of which are strongly visible in the early stages of Y chromosome evolution and are later over-taken by transposable elements in older Y chromosomes. We have also discussed the relationship between microsatellites expansion and other important factors or processes like DNA conformation, (retro)transposition and DNA recombination.

## Materials and Methods

### Fluorescence In Situ Hybridization (FISH)

*Rumex acetosa* seeds were obtained from the seed collection at the Institute of Biophysics, Brno, Czech Republic. The preparation of metaphase chromosomes from root tips of germinating seeds and the protocol for fluorescence *in situ* hybridization and microscopy analysis were performed as described in Kubat et al [20] with slight modifications. We used synthetic oligonucleotide probes that were directly labeled with Cy3 at the 5′ terminus during synthesis by VBC-Biotech (Vienna, Austria). Because of the short length of microsatellite probes used in this study we applied conditions of low stringency in our FISH experiments. Denatured probe (500 ng of DNA) was applied to each slide, covered with a plastic cover slip, and hybridized for 18 h at 37°C. Slides were then washed at room temperature, twice for 5 min each in 2× SSC, twice for 5 min each in 1×SSC and once for 1 min in 1× PBS. To differentiate the Y1 and Y2 chromosomes, the RAYSI tandem repeat (labeled with Spectrum Green) specifically accumulated on the both Y chromosomes, was used as a cytogenetic marker to detect banding pattern differences [45].

### DNA Sequence Analysis

Our 454 sequencing data from *R. acetosa* were obtained using the GS FLX platform (454 Life Sciences, Branford, USA) and can be accessible from SRA under accession numbers SRX118073 and SRX118072. Human continual sequence data were extracted from UCSC Genome Bioinformatics Site (hg19 GCA_000001405.1), whereas our human 454 data came from 1000 genomes project [46]. Human male 454 data can be found under accession NA12814, run SRR006871, human female 454 data can be found under accession NA12815, run SRR006841. *Marchantia polymorpha* male 454 data are deposited under accession SRX030776, run SRR072160. All data were processed using Phobos for discovering microsatellite arrays [47].

Transposable elements positions were searched with a locally installed version of RepeatMasker [48]. The microsatellite - transposable element association was then analyzed in region 50 bp upstream and 50 bp downstream of TE insertion sites found by RepeatMasker with 24% divergence from consensus allowed. In order to identify association of microsatellites with randomly chosen chromosomal loci, we reshuffled positions of all transposons on particular chromosomes using function shuffleBed available in BEDTools package [49], extracted neighborhood (50 bp +−) of these fictitious transposable elements, searched for microsatellites and assessed enrichment. Our association values determined which fraction of transposable elements had at least one microsatellite array in the direct neighborhood (at least 10 bp of an array must be in the distance +−50 bp from insertion site, similarly as area shown on Figure 4).

In Figure S6, we used sex-averaged recombination map from http://www.decode.com/addendum/. Abundance of AC (AC)n A and AAC (AAC)n AA motifs were calculated using 20 kb sliding window for microsatellite arrays longer than 16 bp. Figures with microsatellite distribution were generated using Gnuplot, command-line driven graphing utility available from http://www.gnuplot.info/.

### CD (Circular Dichroism) Spectroscopy

The ability of DNA to adopt an A-DNA or Z-DNA forms was measured by circular dichroism (CD) spectroscopy. CD measurements were done on a Jobin-Yvon CD6 dichrograph in 0.1-cm path-length quartz Hellma cells placed in a thermostated holder (20°C) at a scan rate of 0.5 nm per second. The CD signal was expressed as the difference in the molar absorption Δε of the right- and left-handed circularly polarized light. DNA concentrations were determined on the basis of UV absorption at 260 nm of the sample measured in a 1 mM Na-phosphate buffer with 0.3 mM EDTA, pH 7 at 90°C, using molar extinction coefficients calculated according to Gray et al [50]. UV absorption spectra were measured on a UNICAM 5625 UV/VIS spectrometer (Cambridge, UK). Duplexes were prepared by mixing separate DNA strands in molar concentration 1∶1 and heated to 90°C and slowly cooled to room temperature. Experimental conditions were changed directly in the cells by adding solid CsF or 100% TFE (trifluoroethanol) and the final DNA concentration was corrected for the volume increase. The presence of a positive band at about 260 nm and a negative band at about 210 nm reflects the transition of all oligonucleotides to the A-DNA form at increasing concentrations of trifluorethanol (TFE, Figure S8A, B).

## Supporting Information

Figure S1 **Analysis of microsatellite arrays (all mono-, di- and trinucleotides) using 454 sequencing data in** ***Rumex acetosa*** **.** Number of microsatellite arrays is plotted against array length. Data from male are in blue and from female in red. Counts are calculated per 100 Mb.
(PDF)

Figure S2 **Analysis of microsatellite arrays (all mono-, di- and trinucleotides) using 454 sequencing data in male** ***Marchantia polymorpha*** **.** Number of microsatellite arrays is plotted against array length. Counts are calculated per 100 Mb.
(PDF)

Figure S3 **Analysis of microsatellite arrays (all mono-, di- and trinucleotides) using 454 sequencing data in human.** Number of microsatellite arrays is plotted against array length. Data from male are in blue and from female in red. Counts are calculated per 100 Mb.
(PDF)

Figure S4 **Analysis of microsatellite arrays (all mono-, di- and trinucleotides) in human sex chromosomes.** Number of microsatellite arrays is plotted against array length. The data from the Y chromosome are in blue and from the X chromosome in red. Counts are calculated per 100 Mb.
(PDF)

Figure S5 **Comparison of abundance of all AC (A) and AAC (B) microsatellite arrays (red) and internally located (green) AC and AAC microsatellite arrays from 454 sequencing reads of male** ***Rumex acetosa*** **.**
(PDF)

Figure S6 **Distribution of AC(AC)nA and AAC(AAC)nAA arrays (length >16** **bp) plotted with the profile of recombination rates along human chromosome 1.** Note the absence of both recombination and microsatellites in the (peri)centromeric region.
(JPG)

Figure S7 **The abundance of microsatellite arrays of three monomers or longer using 454 sequencing data in** ***Rumex acetosa*** **, 454 sequencing data of** ***Marchantial polymorpha*** **male, human 454 data and continual sequence of human X and Y chromosomes.** Males or Y chromosome are in blue, females or X chromosome are in red.
(TIFF)

Figure S8 **CD spectroscopy measurement of the ability of (CA)_20_C+(GT)_20_G heteroduplex to adopt an A-DNA form induced by increasing concentration of trifluorethanol (A) and the ability of (CA)_20_C+(GT)_20_G and (CAA)_13_CA+(TGT)_13_TG to adopt Z-DNA (C, D, respectively).** Dependence of B-A transition of all oligonucleotides on TFE concentration monitored by the ellipticity around 265 nm (B). Dependence of B-Z transition of all oligonucleotides on CsF concentration monitored by the ellipticity at 277 nm (E).
(TIF)
