# Supplementary figures and images for: Expansion of Microsatellites on Evolutionary Young Y Chromosome
Source: PLoS One. 2013 Jan 16;8(1):e45519. doi: 10.1371/journal.pone.0045519 (PMC3547029; doi:10.1371/journal.pone.0045519)

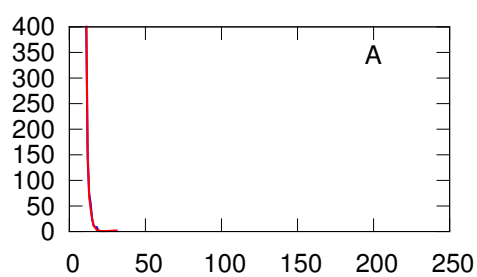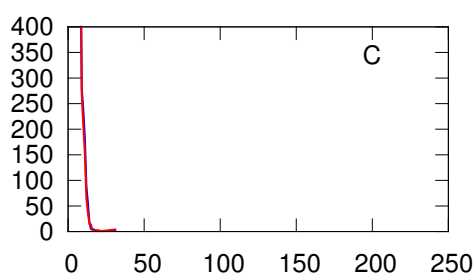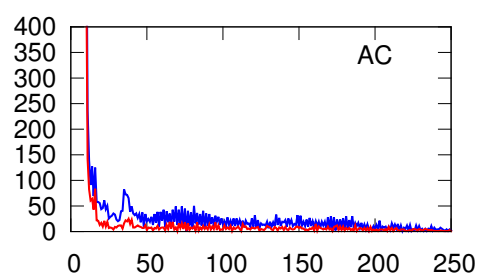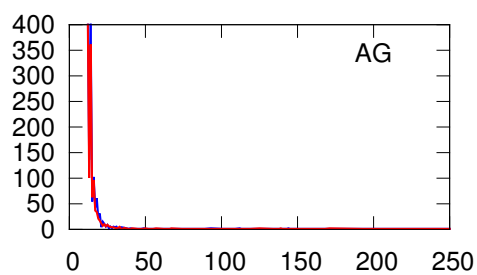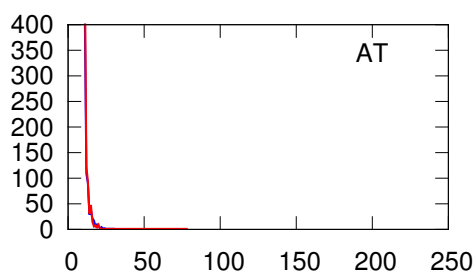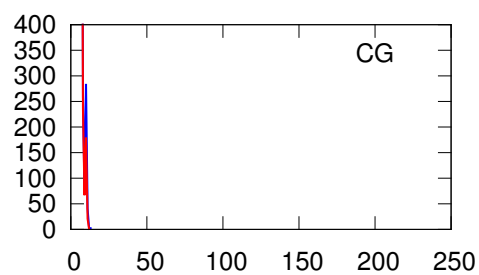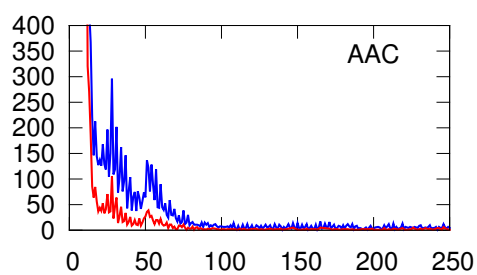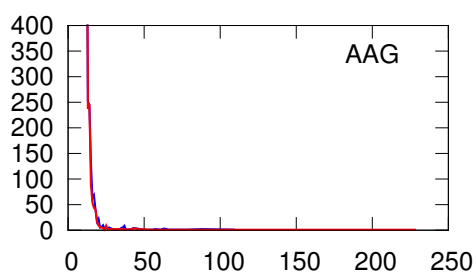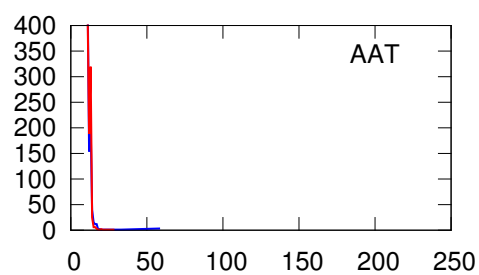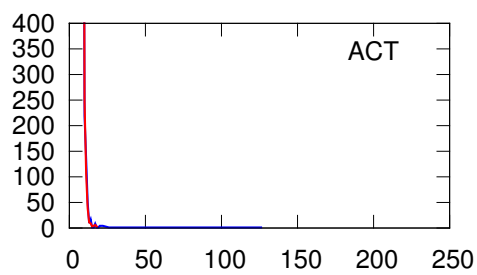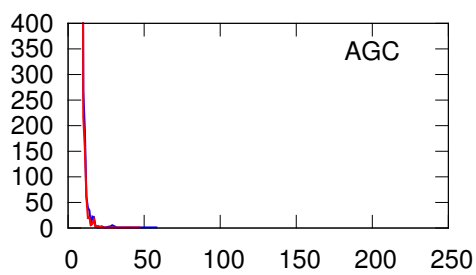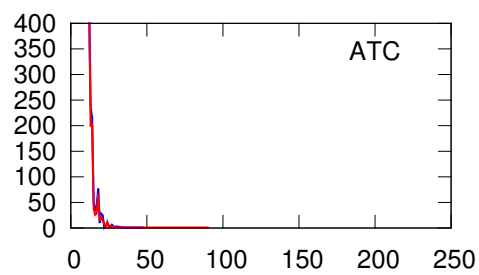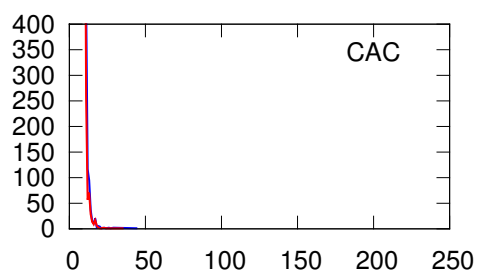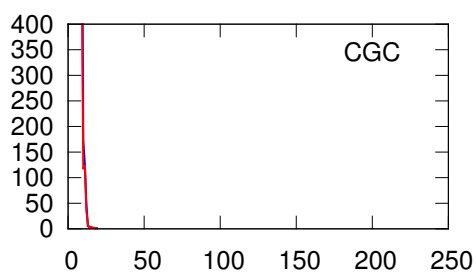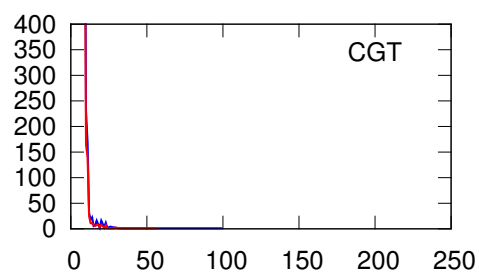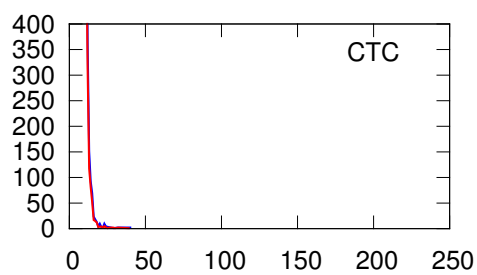

Supplement: Figure S1 — Analysis of microsatellite arrays (all mono-, di- and trinucleotides) using 454 sequencing data in Rumex acetosa . Number of microsatellite arrays is plotted against array length. Data from male are in blue and from female in red. Counts are calculated per 100 Mb. (PDF) [file pone.0045519.s001.pdf]

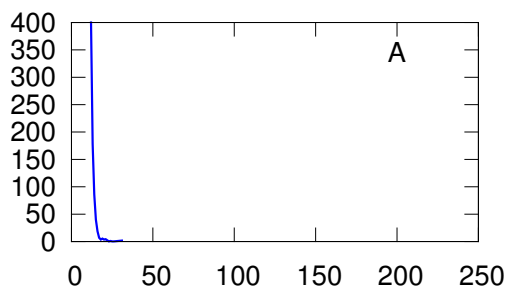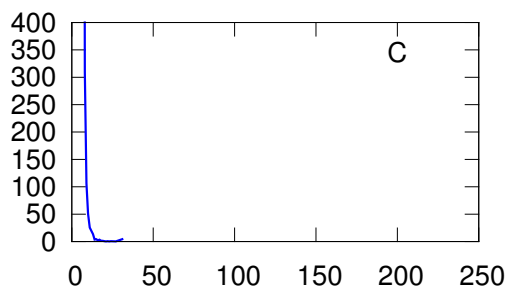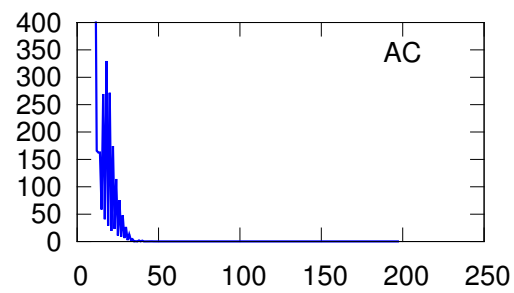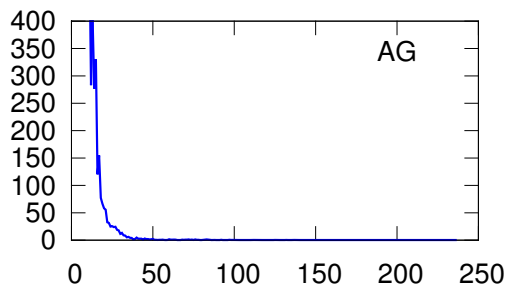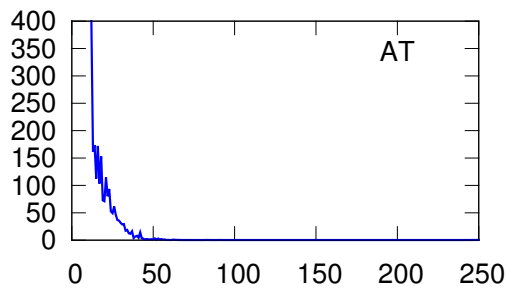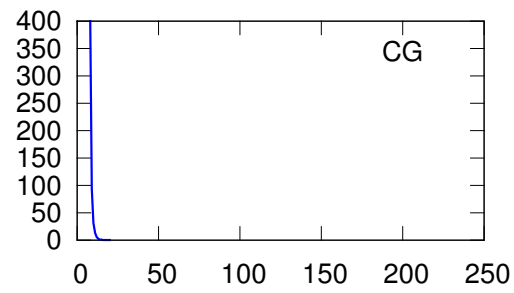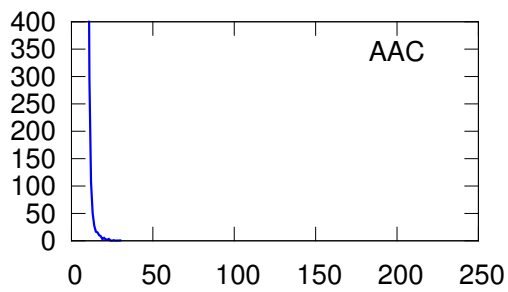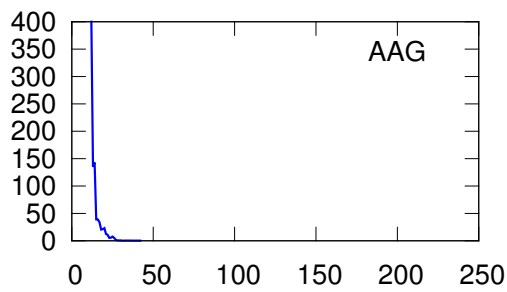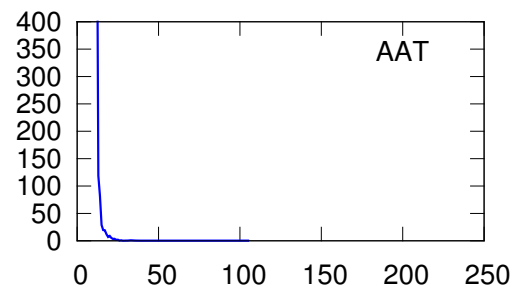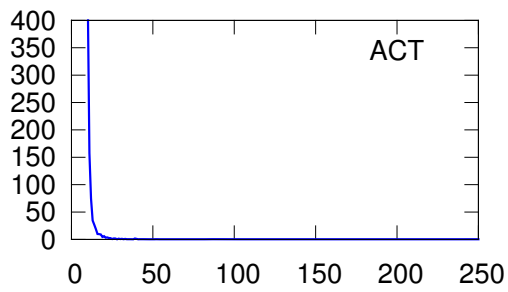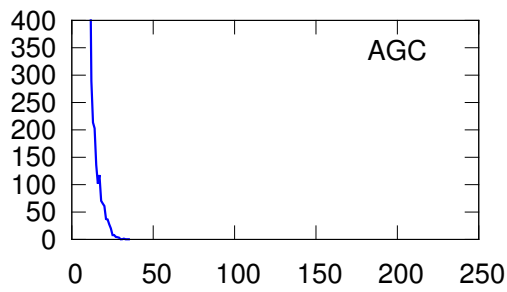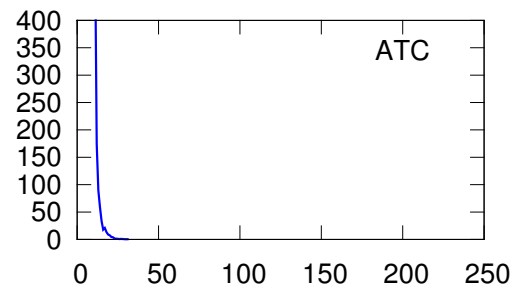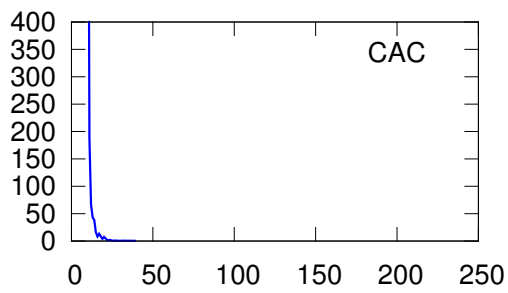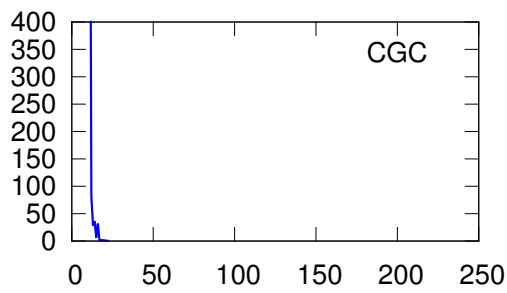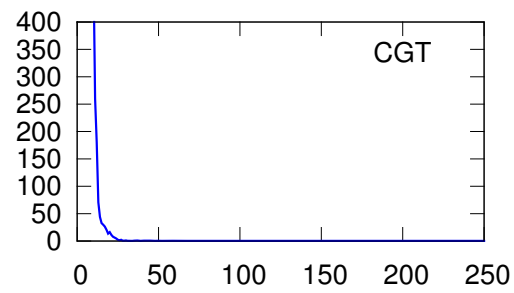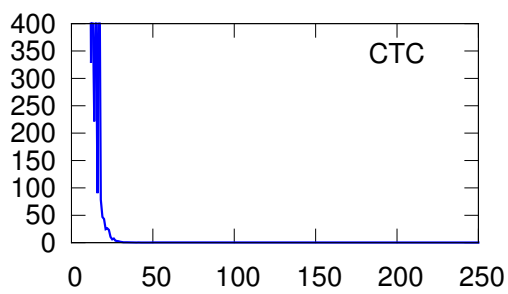

Supplement: Figure S2 — Analysis of microsatellite arrays (all mono-, di- and trinucleotides) using 454 sequencing data in male Marchantia polymorpha . Number of microsatellite arrays is plotted against array length. Counts are calculated per 100 Mb. (PDF) [file pone.0045519.s002.pdf]

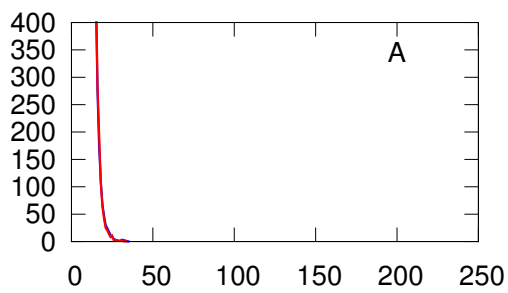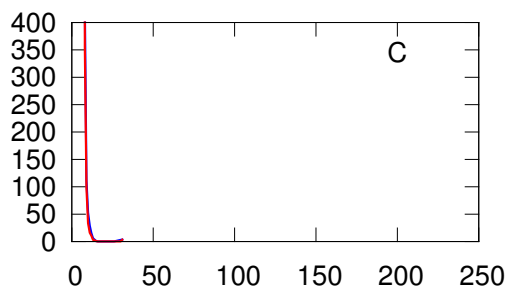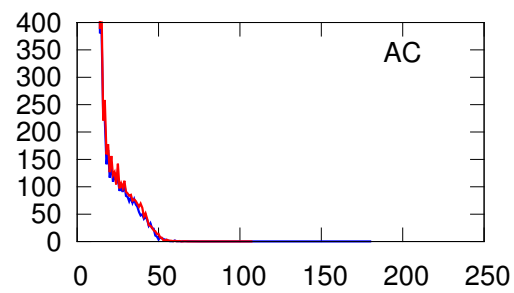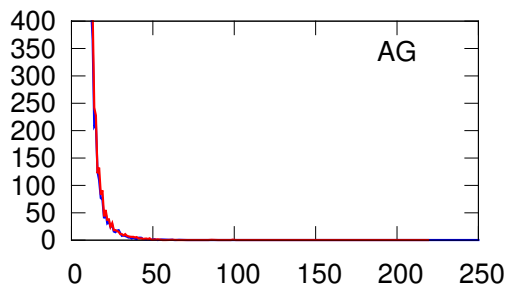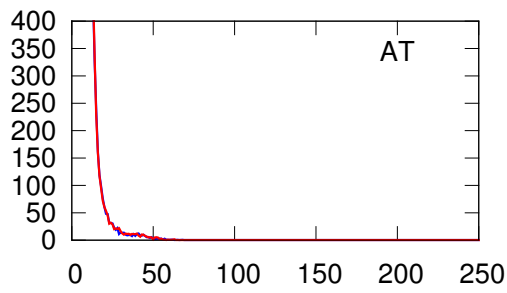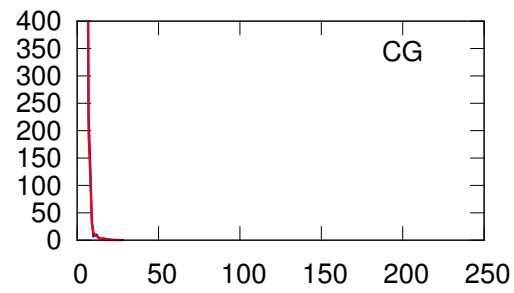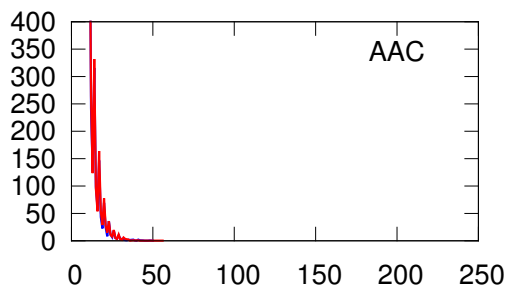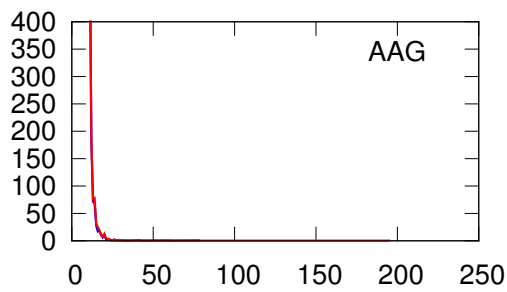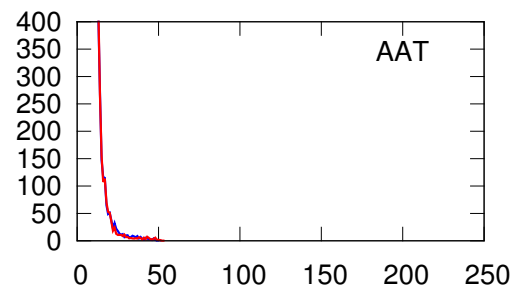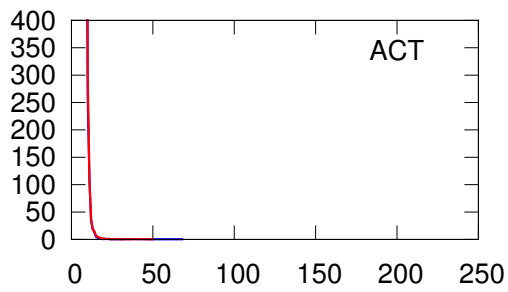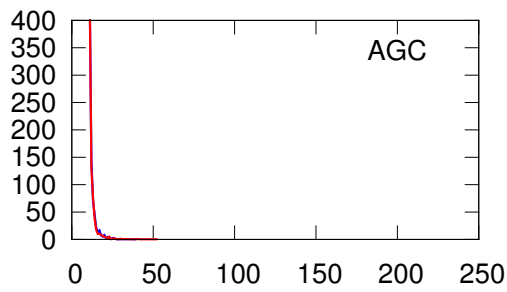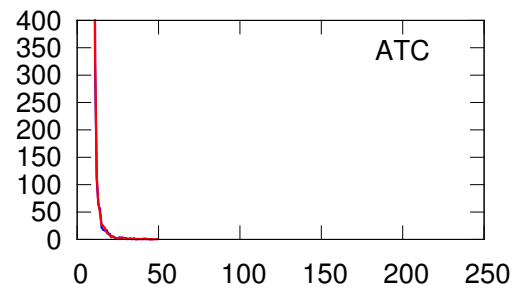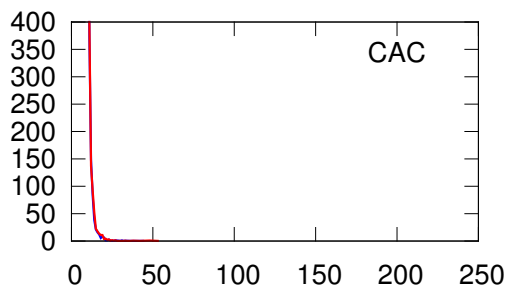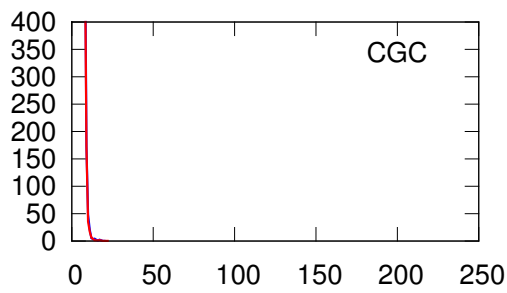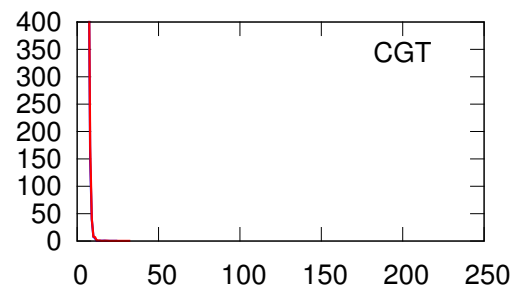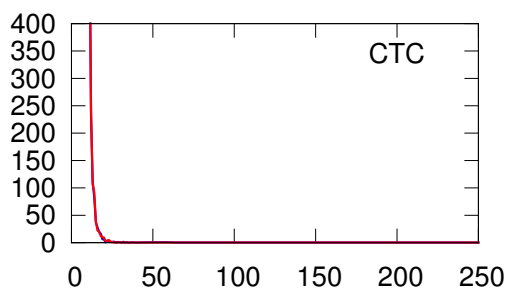

Supplement: Figure S3 — Analysis of microsatellite arrays (all mono-, di- and trinucleotides) using 454 sequencing data in human. Number of microsatellite arrays is plotted against array length. Data from male are in blue and from female in red. Counts are calculated per 100 Mb. (PDF) [file pone.0045519.s003.pdf]

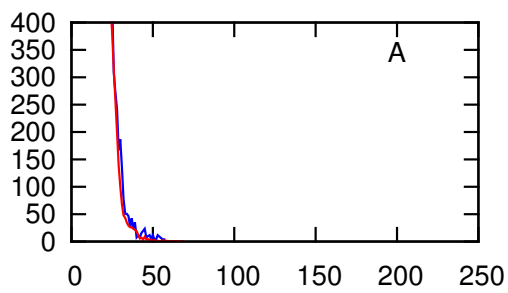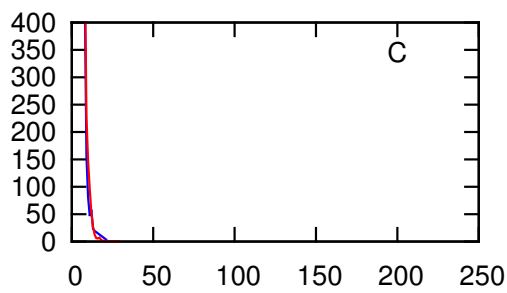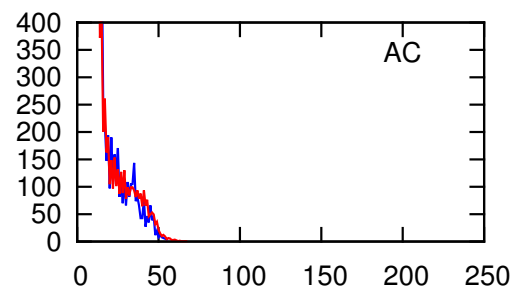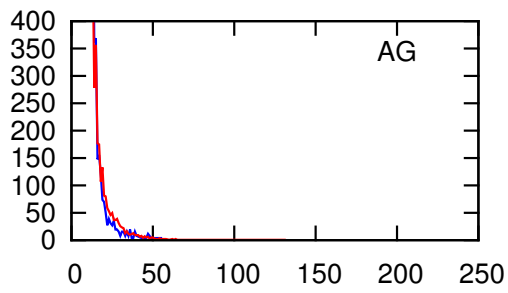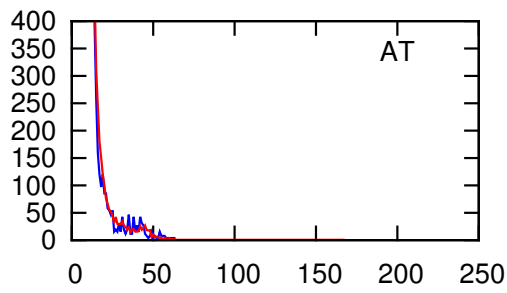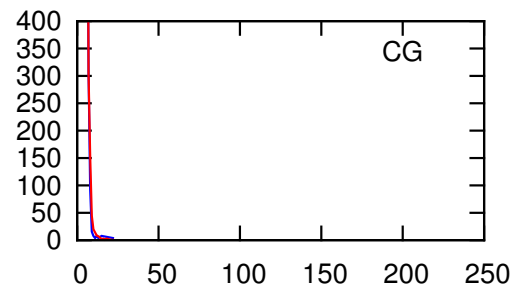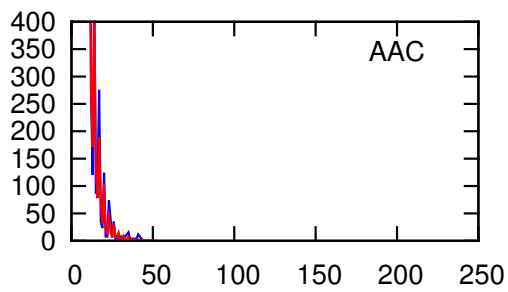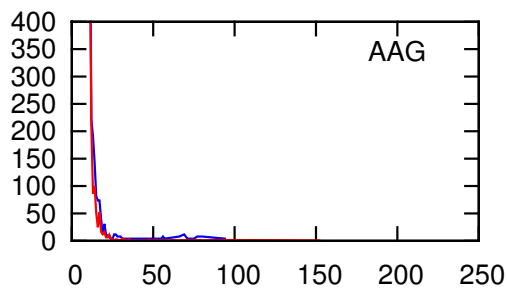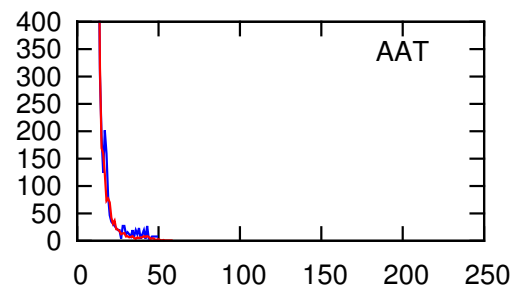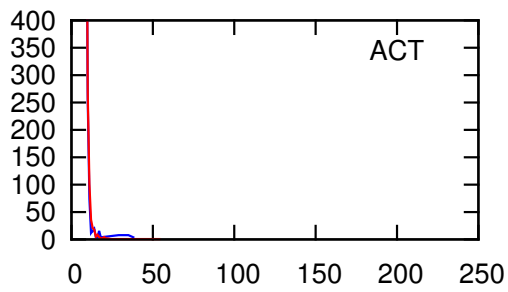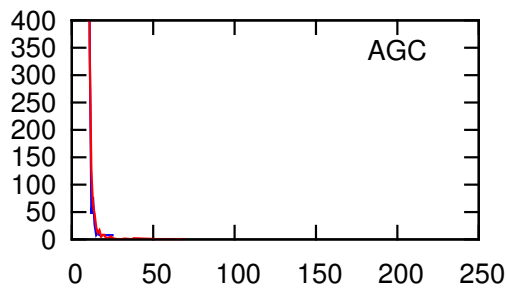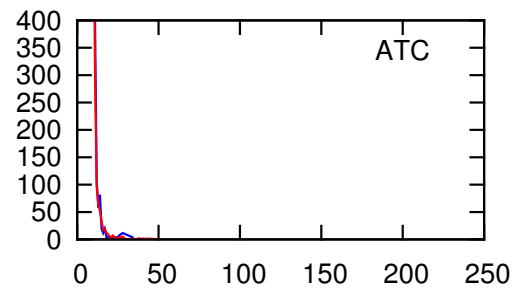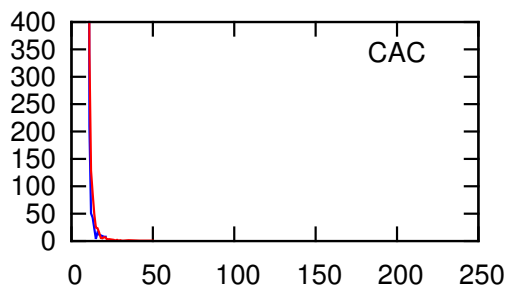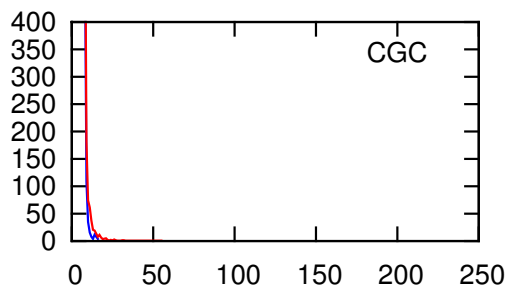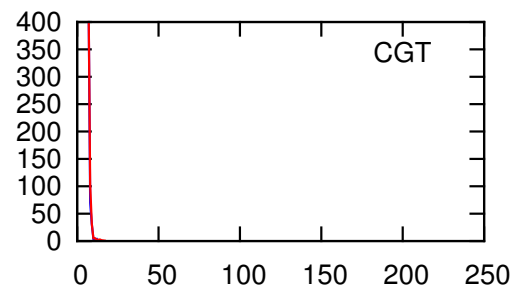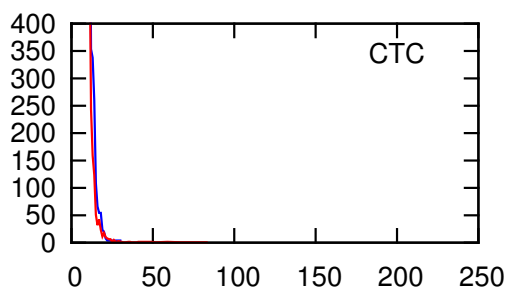

Supplement: Figure S4 — Analysis of microsatellite arrays (all mono-, di- and trinucleotides) in human sex chromosomes. Number of microsatellite arrays is plotted against array length. The data from the Y chromosome are in blue and from the X chromosome in red. Counts are calculated per 100 Mb. (PDF) [file pone.0045519.s004.pdf]

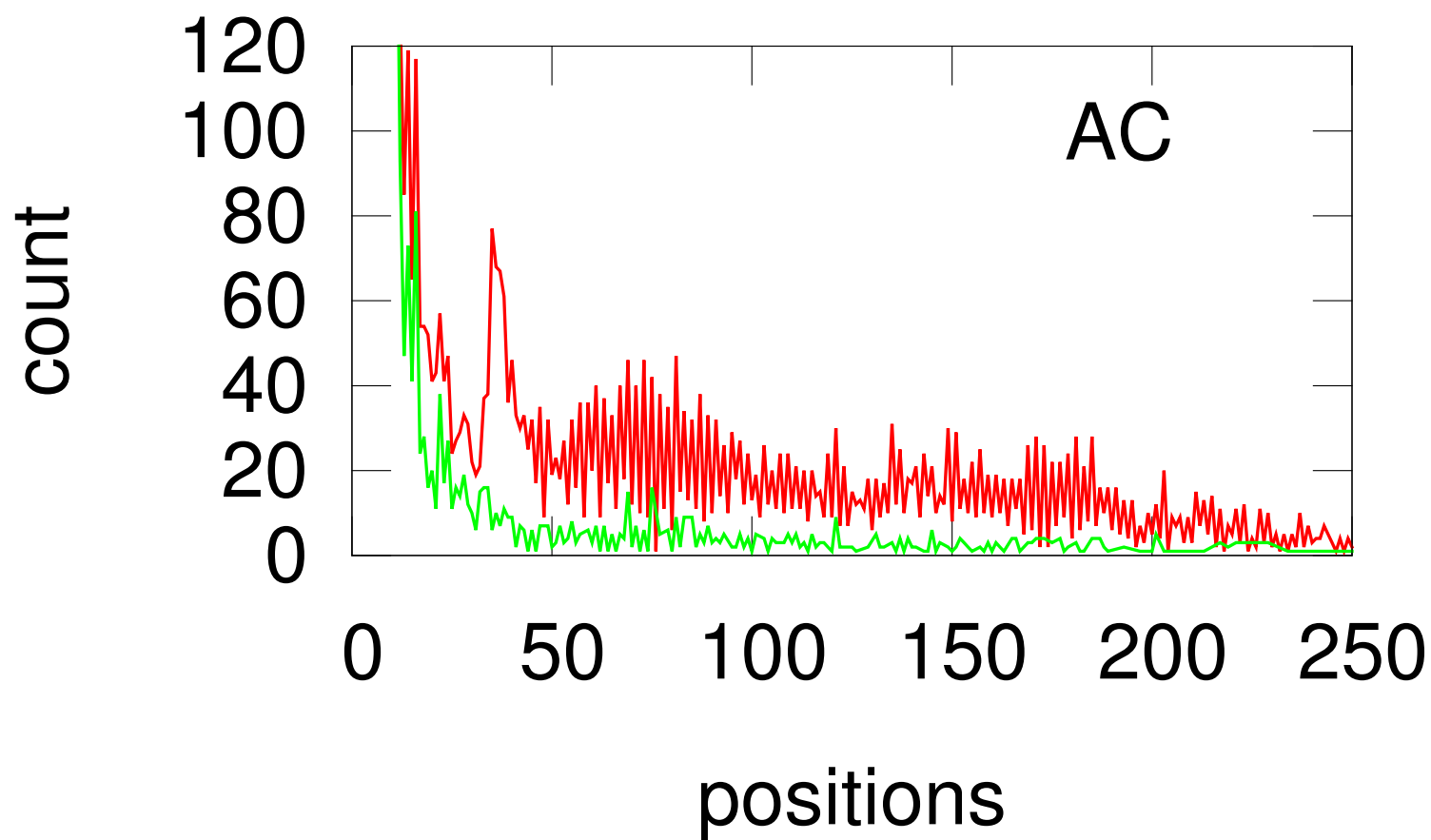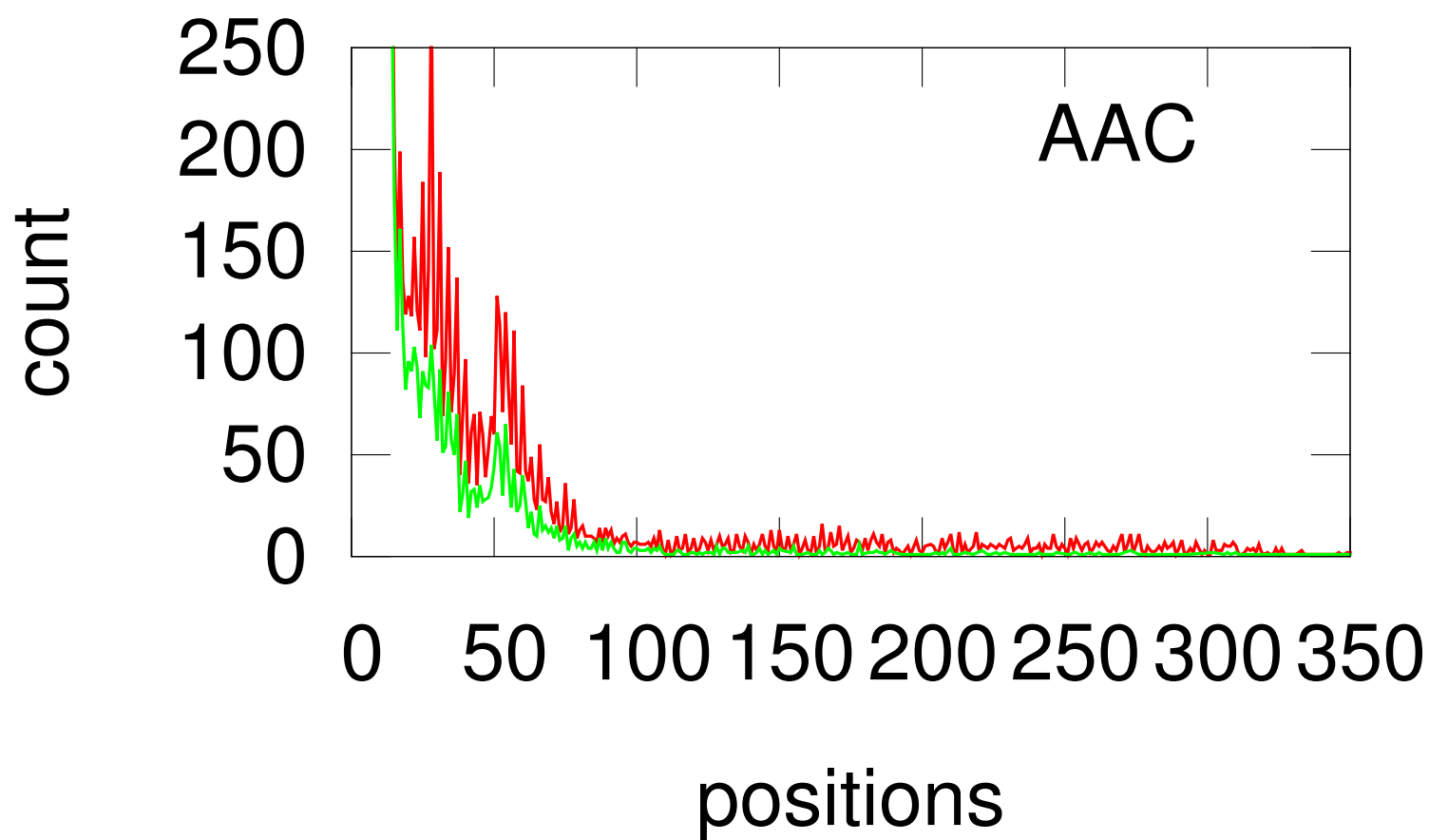

Supplement: Figure S5 — Comparison of abundance of all AC (A) and AAC (B) microsatellite arrays (red) and internally located (green) AC and AAC microsatellite arrays from 454 sequencing reads of male Rumex acetosa . (PDF) [file pone.0045519.s005.pdf]

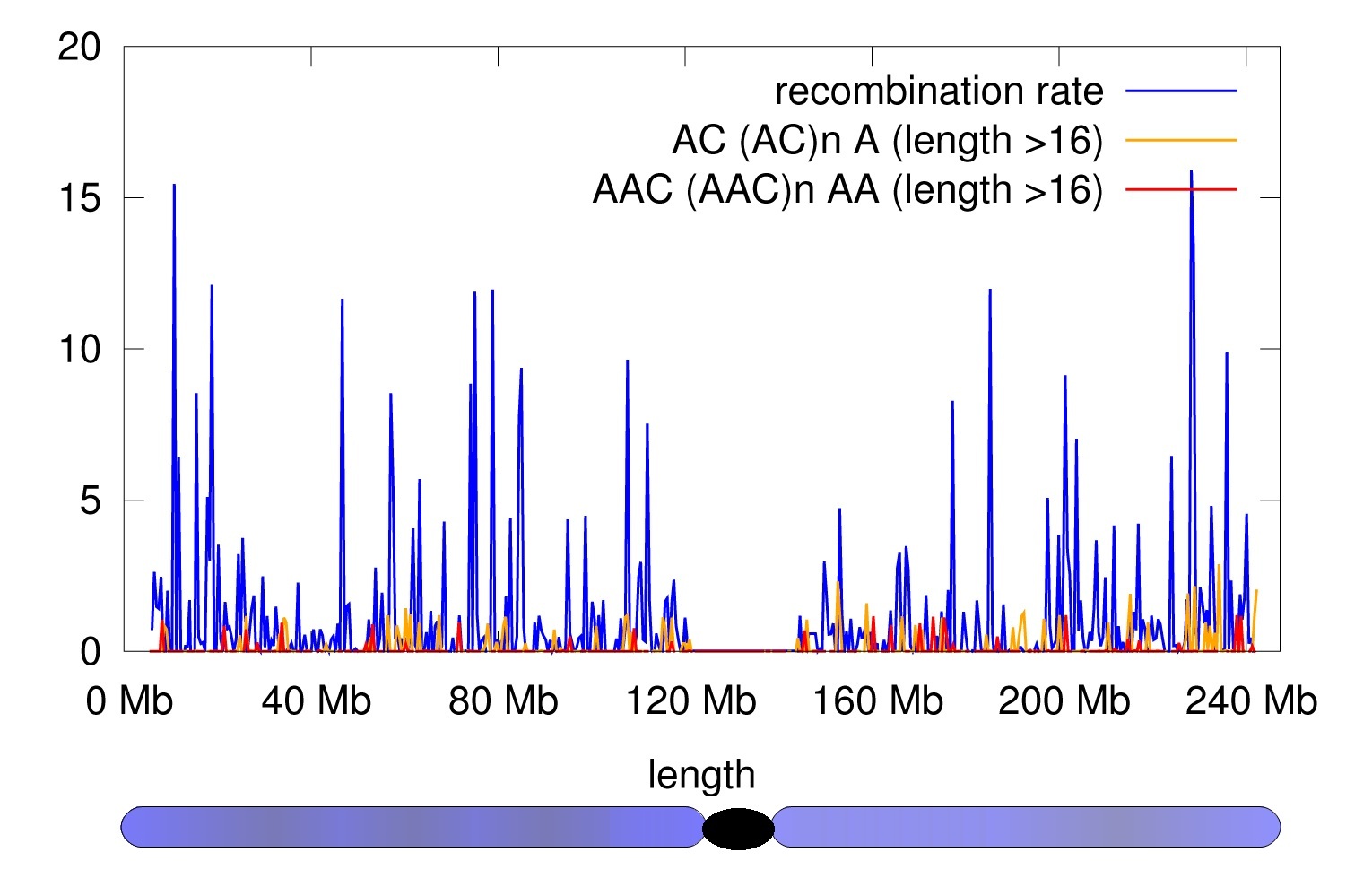

Supplement: Figure S6 — Distribution of AC(AC)nA and AAC(AAC)nAA arrays (length >16 bp) plotted with the profile of recombination rates along human chromosome 1. Note the absence of both recombination and microsatellites in the (peri)centromeric region. (JPG) [file pone.0045519.s006.jpg]

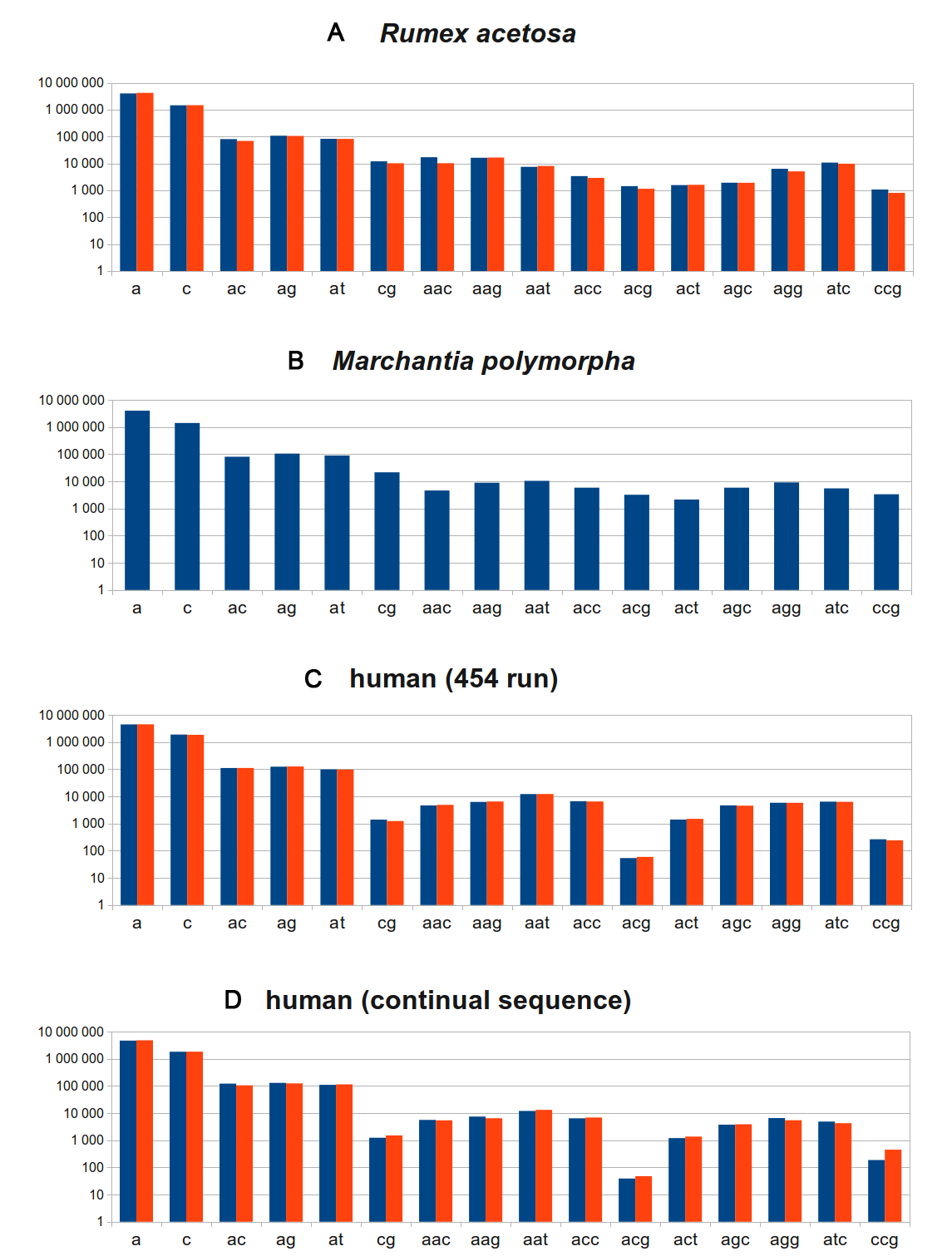

Supplement: Figure S7 — The abundance of microsatellite arrays of three monomers or longer using 454 sequencing data in Rumex acetosa , 454 sequencing data of Marchantial polymorpha male, human 454 data and continual sequence of human X and Y chromosomes. Males or Y chromosome are in blue, females or X chromosome are in red. (TIFF) [file pone.0045519.s007.tif]

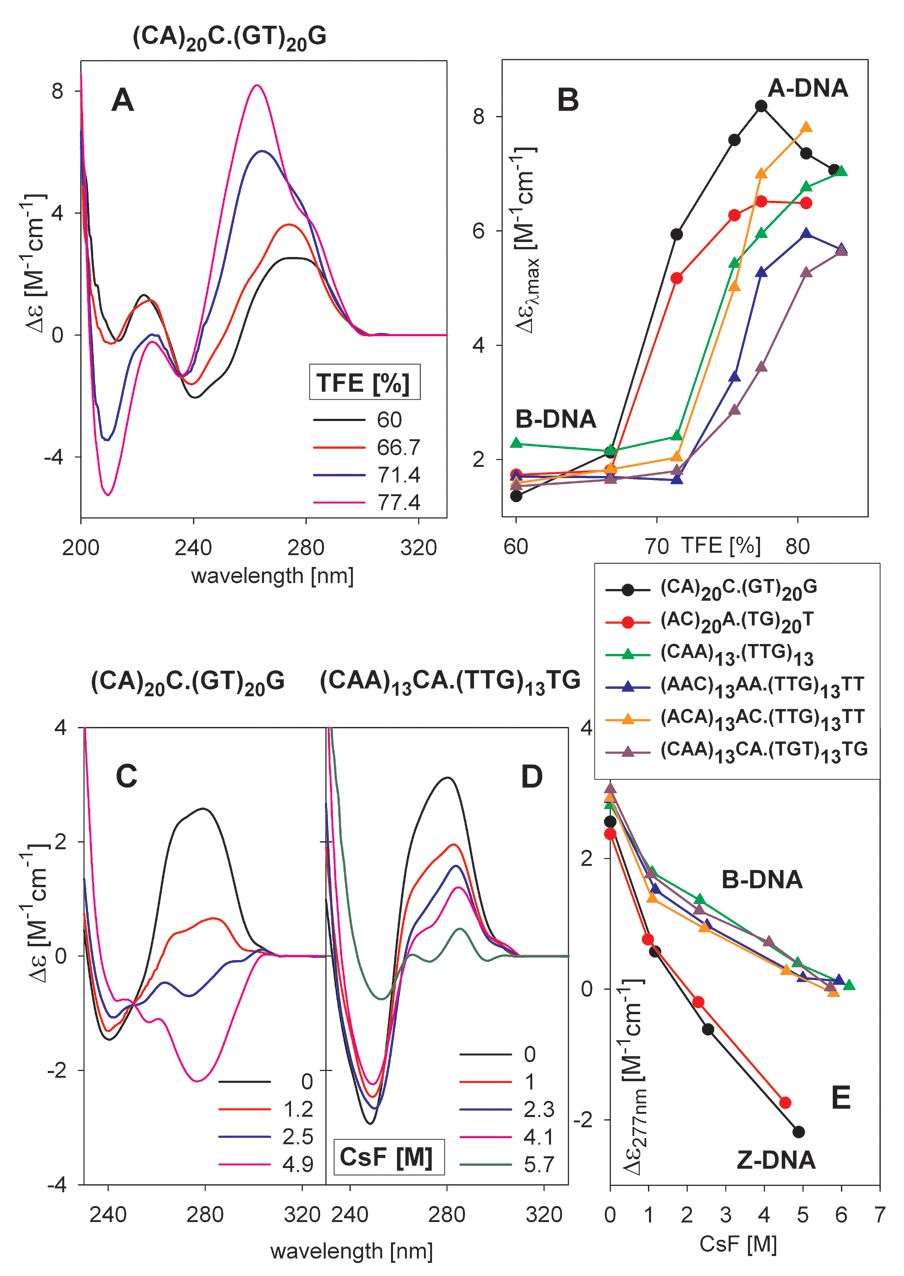

Supplement: Figure S8 — CD spectroscopy measurement of the ability of (CA)20C+(GT)20G heteroduplex to adopt an A-DNA form induced by increasing concentration of trifluorethanol (A) and the ability of (CA)20C+(GT)20G and (CAA)13CA+(TGT)13TG to adopt Z-DNA (C, D, respectively). Dependence of B-A transition of all oligonucleotides on TFE concentration monitored by the ellipticity around 265 nm (B). Dependence of B-Z transition of all oligonucleotides on CsF concentration monitored by the ellipticity at 277 nm (E). (TIF) [file pone.0045519.s008.tif]
